# Human canonical CD157/Bst1 is an alternatively spliced isoform masking a previously unidentified primate-specific exon included in a novel transcript

**DOI:** 10.1038/s41598-017-16184-w

**Published:** 2017-11-21

**Authors:** Enza Ferrero, Nicola Lo Buono, Simona Morone, Rossella Parrotta, Cecilia Mancini, Alfredo Brusco, Alice Giacomino, Stefania Augeri, Antonio Rosal-Vela, Sonia García-Rodríguez, Mercedes Zubiaur, Jaime Sancho, Alessandra Fiorio Pla, Ada Funaro

**Affiliations:** 1 Laboratory of Immunogenetics, Department of Medical Sciences, University of Torino, 10126 Torino, Italy; 2 San Raffaele Diabetes Research Institute, San Raffaele Hospital, 20132 Milano, Italy; 3 Laboratory of Medical Genetics, Department of Medical Sciences, University of Torino, 10126 Torino, Italy; 4 Department of Cellular Biology and Immunology, Instituto de Parasitología y Biomedicina López-Neyra, IPBLN-CSIC, Parque Tecnológico de la Salud de Granada, 18016 Granada, Spain; 5 Department of Life Sciences and Systems Biology, University of Torino, 10123 Torino, Italy

## Abstract

CD157/Bst1 is a dual-function receptor and β-NAD^+^-metabolizing ectoenzyme of the ADP-ribosyl cyclase family. Expressed in human peripheral blood neutrophils and monocytes, CD157 interacts with extracellular matrix components and regulates leukocyte diapedesis via integrin-mediated signalling in inflammation. CD157 also regulates cell migration and is a marker of adverse prognosis in epithelial ovarian cancer and pleural mesothelioma. One form of CD157 is known to date: the canonical sequence of 318 aa from a 9-exon transcript encoded by *BST1* on human chromosome 4. Here we describe a second *BST1* transcript, consisting of 10 exons, in human neutrophils. This transcript includes an unreported exon, exon 1b, located between exons 1 and 2 of *BST1*. Inclusion of exon 1b in frame yields CD157-002, a novel proteoform of 333 aa: exclusion of exon 1b by alternative splicing generates canonical CD157, the dominant proteoform in neutrophils and other tissues analysed here. In comparative functional analyses, both proteoforms were indistinguishable in cell surface localization, specific mAb binding, and behaviour in cell adhesion and migration. However, NAD glycohydrolase activity was detected in canonical CD157 alone. Comparative phylogenetics indicate that exon 1b is a genomic innovation acquired during primate evolution, pointing to the importance of alternative splicing for CD157 function.

## Introduction

CD157 (alias Bst1, ADP-ribosyl cyclase/cyclic ADP-ribose hydrolase 2) is a GPI-anchored glycoprotein, conserved among vertebrates, that belongs to the ADP-ribosyl cyclases (ARCs)^1,2^. The ARC gene family comprises *CD38*, a paralogue of *BST1*, and other more distantly related homologues are found in aplysia, sea urchin and schistosome^1^. For clarity, the nomenclature adopted here will be CD157 to describe the protein whereas gene and transcripts will be denoted with the official HGNC (HUGO Gene Nomenclature Committee) symbol of *BST1*.

Discovered over three decades ago as the Mo5 myeloid antigen^3^, CD157 was identified and characterised as a cell surface receptor expressed in bone marrow stromal cells where it promotes the proliferation of hematopoietic progenitors^4^, hence the name *b*one marrow *st*romal cell antigen *1*. CD157 is highly expressed in monocytes, neutrophils and more immature myeloid stages^5,6^, and in vascular endothelium^7^. CD157 is also expressed in human mesenchymal stem cells (MSC) and is reported to be a receptor for SCRG1 (scrapie responsive gene 1), a protein involved in regulation of MSC self-renewal, migration, and osteogenic differentiation^8^ but also highly expressed in the central nervous system^9^.

We previously showed that CD157 is a key player on orchestrating leukocyte and specific tumor cell type trafficking. CD157 modulates essential neutrophil and monocyte functions such as adhesion to the extracellular matrix (ECM)^5^, cell motility and transmigration^10^. Underlying these functional roles is the capacity of CD157 to bind with high affinity to components of the ECM such as fibronectin, fibrinogen, laminin-1 and type 1 collagen, within their heparin-binding domains^11^. CD157 binding confers outside-in signalling in leukocytes through structural and functional interactions with β_1_^6^ and β_2_-integrins^7,12^.

We also reported that CD157 was expressed in >90% of primary epithelial ovarian cancer (EOC)^13,14^ and in malignant pleural mesothelioma (MPM)^15^. In these specific cancer types, stemming from tissues with a common embryonic origin, high expression of CD157 correlates significantly with increased tumor aggressiveness and is an independent prognostic factor for overall survival in EOC and in biphasic MPM^13,14^. CD157 is widely expressed in M4 and M5 subtypes of acute myeloid leukaemia (AML)^3^, notably in the patient group characterised by adverse prognosis according to the European LeukemiaNet (ELN) classification and CD157 is currently under investigation as a target for antibody-based immunotherapy in AML^16,17^. CD157 is also a promising marker for disease monitoring in B-cell precursor acute lymphoblastic leukaemia (BCP-ALL) where high CD157 expression on malignant cells discriminates them from CD157-negative normal B cells from the same patient^18^.

While the receptor role of CD157 has been clearly delineated, the functional significance of CD157 as an enzyme is less clear at present. Human CD157 is reported to have ADP-ribosyl cyclase activity at acidic pH (4.0–6.5) and in the presence of metal (Zn^2+^ and Mg^2+^) ions^19,20^, but its quantitative contribution to synthesizing the potent intracellular second messenger cyclic adenosine 5′-diphosphoribose (cADPR) by β-NAD^+^ conversion is currently unknown. In contrast, the NAD^+^ glycohydrolase (EC 3.2.2.5) activity of CD157 is readily detectable^20,21^ and leads to release of adenosine 5′-diphosphoribose (ADPR) which can act both as a substrate and as a messenger^22^. In contrast to the observations made in human models, in mouse there is evidence that cADPR generation by CD157 may be biologically relevant in mediating paracrine signalling in hematopoiesis^23^, in gut stem cell signalling in response to calorie restriction^24^ and in social behaviour^25,26^, envisaging a role for CD157 as a neuro-entero-immune regulator^2^. A further neuroregulatory role for human CD157 is under investigation following genome-wide association studies that have linked *BST1* polymorphisms to susceptibility for Parkinson’s disease^27,28^. Parkinson-like behavioural abnormalities have been detected in *bst1/bp3* knockout mice which show signs of non-motor dysfunction such as anxiety and depression-like symptoms^29^.

The human *BST1* gene and cDNA were characterised in the 1990s using standard molecular cloning procedures^4,30^ with *BST1* described as a 9-exon gene like its murine counterpart *bst1/bp3* ^31^. *BST1* maps to chromosome 4p15, next to its paralogue or gene duplicate *CD38* ^32^. *BST1* and *CD38* ARC genes show conservation in their gene structure and protein sequence^1^. All the current functions of CD157 have been attributed to the only form known to date, the canonical protein of 318 amino acids believed to be derived by constitutive splicing of the 9-exon *BST1* gene. However, in preparation for analysis of human *BST1* transcript expression in normal human tissues by PCR, a primer pair designed to amplify a target sequence spanning exons 1 and 2 unexpectedly yielded two bands instead of one. As we are not aware of other forms of CD157 beyond the canonical, we investigated the PCR result which led to an upheaval in *BST1*/CD157 proteogenomics and we report here that: (i) by finding of a previously undescribed exon, exon 1b, we redefine *BST1* as a 10-exon gene; (ii) there is a second *BST1* transcript expressed in neutrophils and other tissues which encodes a novel and functionally divergent proteoform of 333 amino acids, CD157-002; (iii) production of canonical CD157, or CD157-001, requires exclusion of exon 1b by alternative splicing; and (iv) that exon 1b is a dynamically evolving exon present in phylogeny in *BST1* orthologous genes in primates.

## Results

### THP1 monocytic cell line and PMN express a *BST1* transcript with a novel exon

**Figure 1:**
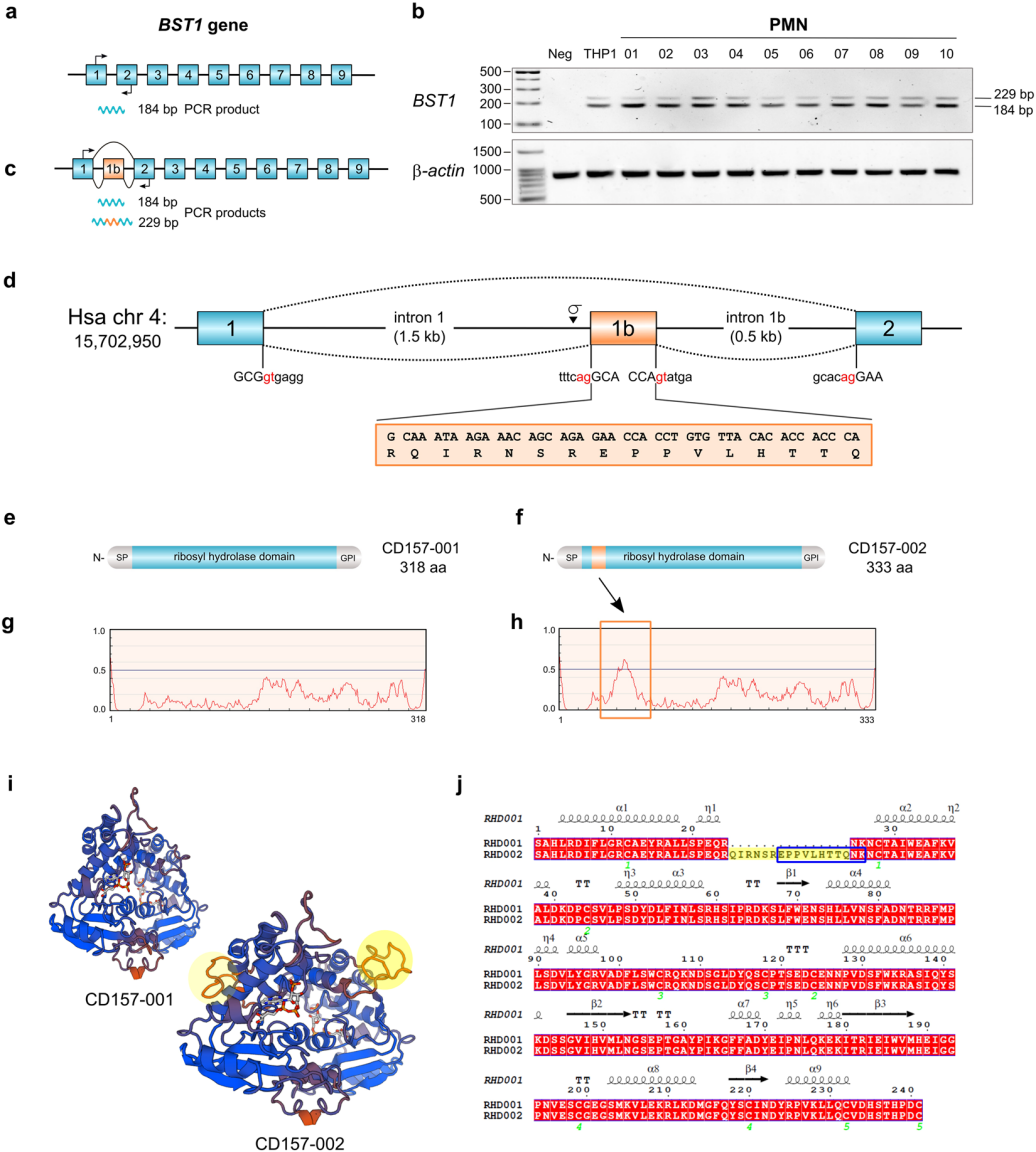
Identification and characterization of human *BST1* exon 1b. (**a**) Canonical 9-exon version of human *BST1* showing the 184 bp amplicon expected by RT-PCR priming on exon 1 (arrow, forward primer) and exon 2 (arrow, reverse primer). (**b**) Agarose gel showing that THP1 cells and PMN from ten healthy donors yield doublet transcripts by RT-PCR. (**c**) Revised human *BST1* gene structure with 10 exons following inclusion of a novel exon, 1b, identified experimentally by RT-PCR. The canonical transcript *BST1-*001 is alternatively spliced, skips exon 1b, and yields the 184 bp amplicon by RT-PCR; transcript *BST1-*002 is constitutively spliced, includes exon 1b, and yields the 229 bp amplicon by RT-PCR. (**d**) Schematic representation of the *BST1* genomic locus on *Homo sapiens* chromosome 4 showing intron/exon boundaries of exon 1, 1b and 2. The orange box below exon 1b shows the 15 aa sequence added in-frame to the CD157 polypeptide by exon 1b inclusion. Consensus GT/AG splice sites are depicted in red. (**e**,**f**) Schematic models of the CD157 isoform polypeptides showing location of signal peptide (SP), *ribosyl hydrolase* domain (RHD) and GPI-anchoring propeptide in canonical CD157-001 (318 aa) and CD157-002 (333 aa) proteins, the latter including the exon 1b-encoded insert (orange band). (**g**,**h**) Results of comparative analysis of CD157 polypeptides by IUPRED which predicts intrinsic disorder. A tract of notable difference between CD157 proteofroms is boxed, corresponding to the region where exon 1b is included in CD157-002. (**i**) View of molecular models of CD157 dimer crystal structure PDB 1ISI (NCBI Structure): in background, CD157-001 dimer; in front, SWISS-MODEL-predicted dimer of CD157-002 with exon 1b-encoded peptides (both monomers) enclosed in yellow circles. (**j**) Alignment of the RHD from canonical CD157-001 and CD157-002. Secondary structures (alpha helices, beta sheets and turns) are indicated. Numbers in green show cysteine residues involved in disulphide bridge formation. RHD-002 sequence highlighted in yellow shows the 15 aa insert encoded by exon 1b, and peptide boxed in blue was identified in the proteomics database www.proteomicsdb.org.

In preparation for analysis of the distribution of *BST1* transcripts in human tissues by RT-PCR, an oligonucleotide primer pair was selected based on the orthodox 9-exon structure of the human *BST1* gene, designed to amplify a 184 bp fragment spanning exons 1 and 2 of the canonical *Bst1* transcript (*BST1-001*; Ensembl ENST00000265016; GenBank NM_004334.2) (Fig. 1a). THP1, a CD157-positive human monocytic leukaemia cell line, was selected as test template. Alongside the expected band, an additional faint upper band >200 bp long was observed (see the THP1 sample, Fig. 1b). To exclude a cell line artifact, the primer pair was tested on cDNA samples obtained from freshly isolated polymorphonuclear leukocytes (PMN) from 10 healthy adults, as PMN also show high expression of CD157^5^. By PCR, all PMN samples showed the strong lower band/faint upper band couplet (Fig. 1b).

After agarose gel electrophoresis, the upper band was isolated and its sequence obtained, revealing it to consist of 229 bp. Although the sequence contained the expected 184 bp fragment, this was interrupted at the exon 1/exon 2 junction by inclusion of a 45 bp insert (Fig. 1c). As this 45 bp sequence appeared to represent a previously unrecognized exon, the Ensembl human database (GRCh38.p7) was analysed and a second *BST1* transcript (*BST1-*002; ENST00000382346) was indeed found which included an exon formed by the same 45 bp sequence. This previously uncloned exon was designated exon 1b to maintain the numbering of homologous exons 2–9 shared with other vertebrate *BST1* genes. Exon 1b is located within the *BST1* gene on human chromosome 4p15, between exon 1 (1,587 bp downstream of the 3′ end) and exon 2 (550 bp upstream of the 5′end) (Fig. 1c,d; Supplementary Figure S1), explaining the results obtained by PCR (Fig. 1c). The 45 bp sequence has all the hallmarks of an exon^33^: it is flanked by canonical AG/GT splice sites, shows a putative branch point (5′-TGCTGAT-3′) 24 bp upstream of the intron 1 splice acceptor site and a polypyrimidine tract (Fig. 1d; Supplementary Figure S1). Accordingly, the structure of human *BST1* has been revised to that of a 10-exon gene (Fig. 1c).

Using either exon 1- or exon 1b-specific forward primers with a common exon 9 reverse primer, RT-PCR confirmed that THP1 and PMN have two full-length *BST1* isoform transcripts which correspond to the two transcripts described in the Ensembl database. These transcripts are: (i) alternatively spliced *BST1-001* that skips exon 1b and encodes the canonical form of CD157 with 318 amino acids (Fig. 1e) and (ii) constitutively spliced *BST1-002* (GenBank acc. no. 1747489) in which exon 1b is inserted in-frame to give an open reading frame encoding a predicted polypeptide of 333 aa, comprehensive of a signal peptide, complete *ribosyl_hydrolase* domain (Pfam 002267), conserved N-linked glycosylation sites and disulfide-forming cysteines, and C-terminal GPI pro-peptide (Fig. 1f). This proteoform is designated CD157-002. The 15 aa insert is located towards the N-term of the *ribosyl-hydrolase* domain (RHD). Analysis of the 318 and 333 aa variants by IUPRED, a software program that predicts ordered and disordered regions in proteins^34^, suggests that the 15 aa insertion constitutes an intrinsically disordered region, while the remaining structured region of the RHD remains basically unchanged in CD157-002 with respect to canonical CD157 (CD157-001) (Fig. 1g,h). To date, only the 3D structure of canonical CD157 dimers is available; based on one of these structures (PDB) we have modelled the predicted CD157-002 3D structure (Fig. 1i), using the Swiss-model program^35^. Exon 1b would be inserted after Arg63 in a 3_10_ helix, *i*.*e*., a C-term extension of the α1 helix (Fig. 1j).

Finally, the RNA-based evidence was corroborated by peptide evidence indicating production of CD157-002 protein *in vivo*: a tryptic peptide (EPPVLHTTQNK) encoded by exon 1b (aa 1–9 of the peptide) and exon 2 (aa 10–11) was identified in the www.proteomicsdb.org database as belonging to the 333 aa form of CD157 (acc. no. A6NC48). The peptide is shown boxed within the CD157-002 sequence in Fig. 1j.

### Comparative expression of *BST1-001* and *BST1-002* transcripts

**Figure 2:**
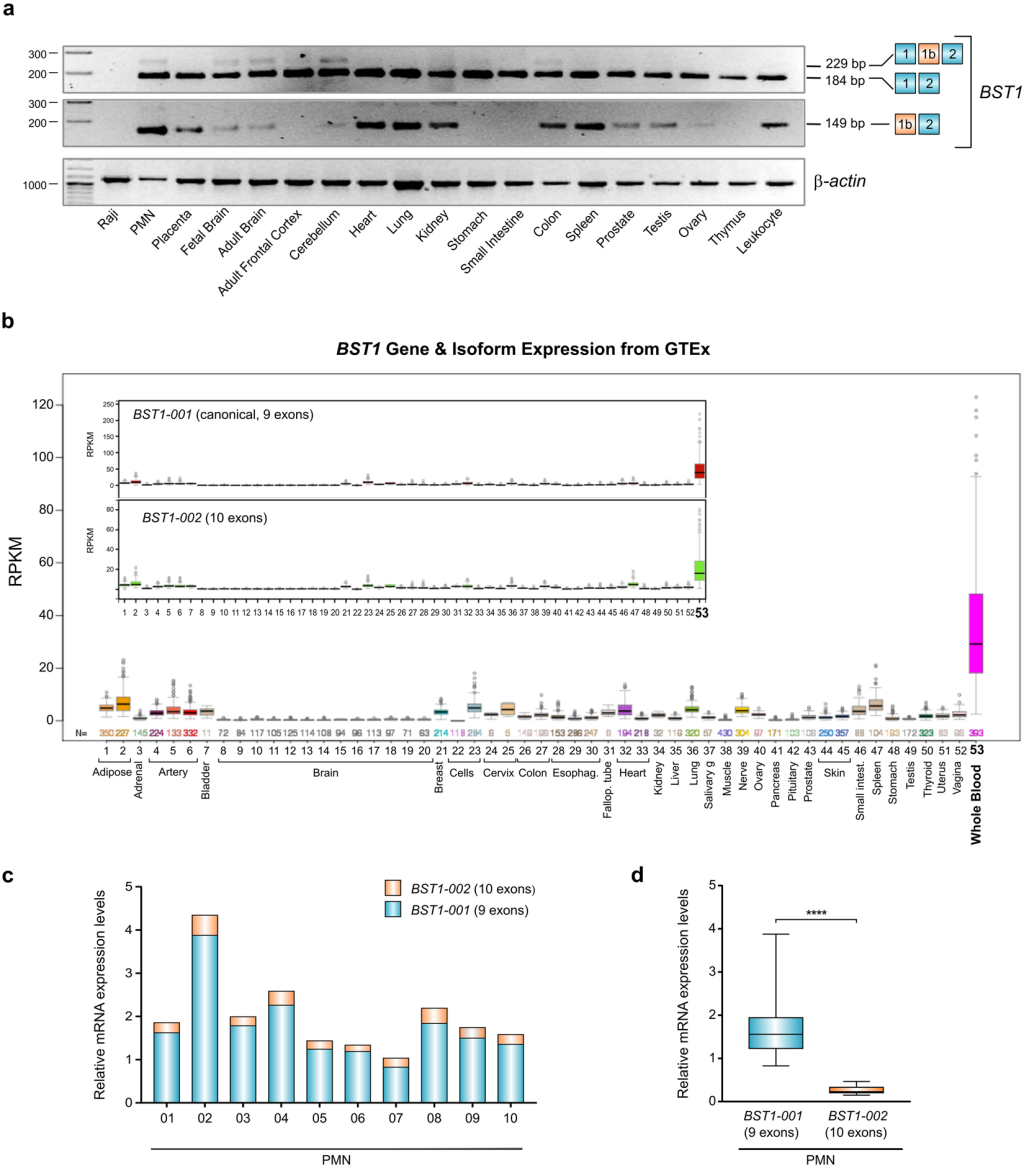
Expression profiles of *BST1-001* and *BST1-002* in human tissues. (**a**) RT-PCR analysis of *BST1* expression in a multiple normal human tissue RNA panel. Amplified exons are indicated schematically on the right. β-actin is the internal control. A composite of representative gels is shown, within which each tissue type is indicated. Full-length gels are presented in Supplementary Figure 3. (**b**) Cross-tissue comparison of *BST1* expression in 53 nonpathological human tissues from the GTEx resource. Expression is measured by RNA-seq and presented as Reads Per Kilobase transcript per Million reads (RPKM). The inserts show *BST1* expression broken down into the *001* and *002* components. (**c**) Relative mRNA levels of *BST1-001* and *BST1-002* transcripts by RTqPCR expression analysis. The same cDNA amount of *BST1-001* and *BST1-002* was added to both assays. The threshold of *GUSB* was used to determine the ratio of each transcript. The relative expression of each transcript was calculated as the percentage of the sum of *BST1-001* + *BST1-002*. Bars represent *BST1-001* versus *BST1-002* ratio, expressed as mean of three independent experiments. (**d**) Box plots showing expression of the two *BST1* transcripts in PMN. Boxes indicate the range (25^th^–75^th^ percentiles), whiskers indicate major and minor values, and the horizontal lines within the boxes indicate the median expression level of *BST1-001* and *BST1-002* transcripts. Results are expressed as relative BST1 mRNA variants expression in PMN from healthy subjects (****p < 0.0001, Student’s t test).

As was originally planned, *BST1* transcript expression was studied by RT-PCR in a panel of human tissues, only now the experiments were carried out using the exon 1/exon 2 primer pair that could simultaneously detect *BST-001* and *-002* transcripts (Fig. 2a). *BST1-001* transcript was detected in all samples examined here except for Raji Burkitt’s lymphoma cell line whereas the *BST1-002* transcript containing exon 1b was most evident in PMN, fetal and adult brain, cerebellum and colon. Using an exon 1b-specific forward primer, *BST1-002* was easily detected not only in PMN but also in placenta, heart, lung, kidney, colon and spleen (Fig. 2a).

The Genotype-Tissue Expression (GTEx) portal (http://www.gtexportal.org) offers a panoramic view of *BST1* transcript expression in 53 nonpathological human tissues. According to this dataset, the *BST1* gene is most highly transcribed in whole blood (presumably in myeloid cells, as erythroid and lymphoid cells are CD157-negative), with expression in other human tissues dwarfed in comparison (Fig. 2b). GTEx data was also available for selective expression of the canonical and *BST1-002* isoforms, with *BST1-001* the more expressed of the two (Fig. 2b, insets).

Therefore PMN were selected as the representative cell population to compare relative expression of *BST1-001* and *BST1-002* isoform transcripts by quantitative PCR (qPCR). Analysis of PMN RNA from 10 healthy adults showed remarkable inter-donor variability in the amount of both *BST1-001* and *002* transcripts (Supplementary Figure S2), and that the *BST1-001* transcript was always more highly expressed than *BST1-002*, consistently representing ~75–80% of the sum of the two transcripts (Fig. 2c,d).

### Comparative functional analysis of CD157-001 and CD157-002 expressed in HeLa cells

**Figure 3:**
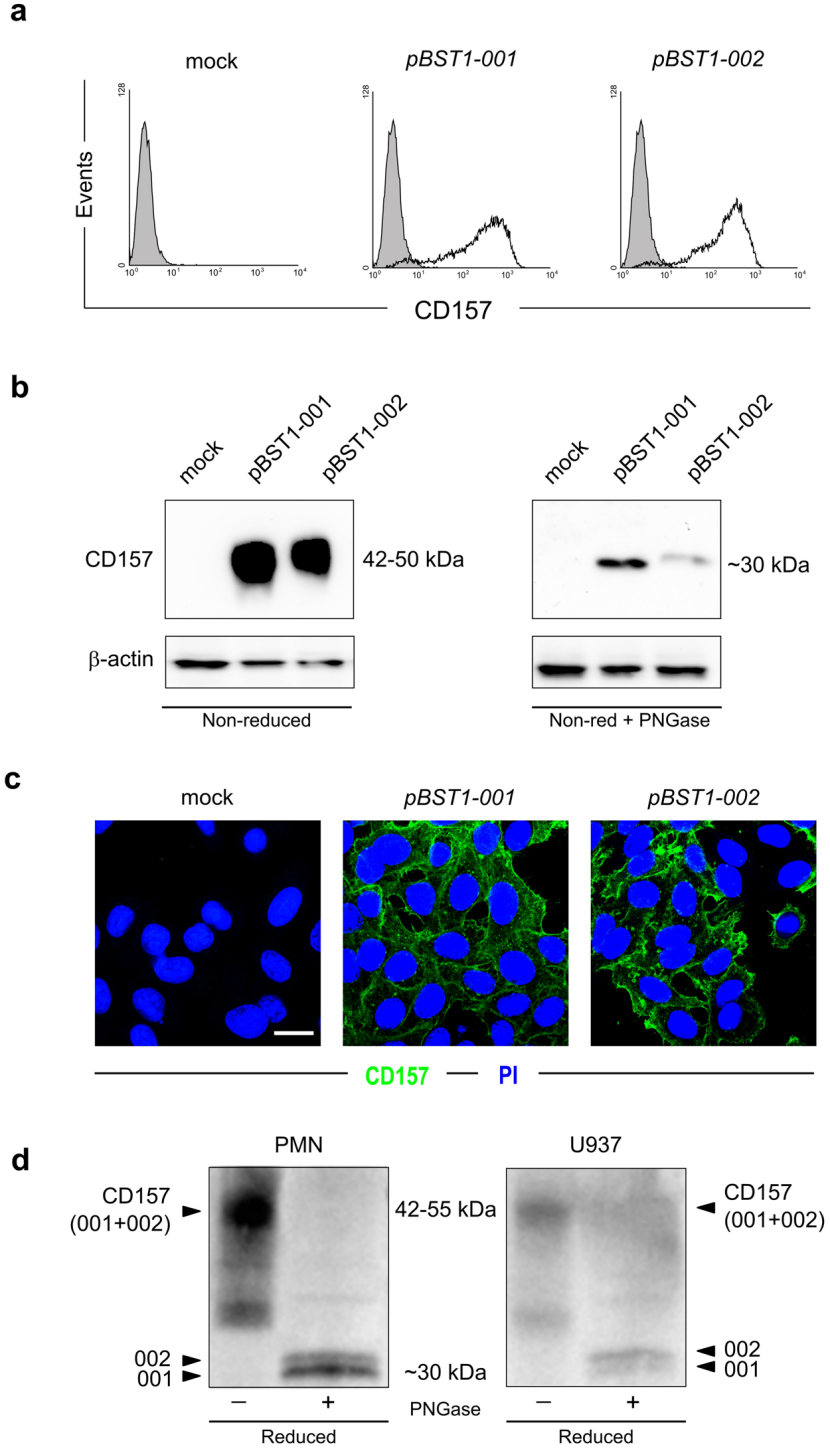
Comparative analyses of CD157 proteoforms expressed in HeLa cells and PMN. (**a**) Flow cytometric analysis of the expression of canonical CD157 (*pBST1-001*) and CD157-002 (*pBST1-002*) in HeLa cells. In each sample, 10^4^ cells were analysed; x-axis = fluorescence intensity, y-axis = number of cells (events). Shaded histograms indicate isotype-matched control IgG, white profiles represent expression of the indicated surface molecules in mock, *pBST1-001* and *pBST1-002* transfectants, respectively. (**b**) Western blot analysis of CD157-001 and -002 expressed in HeLa cells. Left panel, after SDS-PAGE in non-reducing conditions, detected with anti-CD157 mAb (SY/11b5); right panel, samples were treated treated with PNGase to remove N-linked oligosaccharides; full-length blots are presented in Supplementary Figure 4 (top panel). (**c**) Representative immunofluorescence images of the membrane localization of CD157-001 and CD157-002 proteins (in green) in HeLa cells transfected with expression vector of the two different *BST1* isoforms. Nuclei were counterstained with propidium iodide (in blue). Cells were analysed using an Olympus FV300 laser scanning confocal microscope and were imaged using a 60X oil immersion objective (1.4 NA). *Scale bar*: 10 μm. (**d**) Western blot analysis of CD157-001 and -002 expressed in PMN (left panel) or U937 (right panel) after SDS-PAGE in reducing conditions, detected with polyclonal anti-CD157 IgG; where indicated, samples were treated with PNGase to remove N-linked oligosaccharides. One representative experiment is shown (n = 3). Full-length blot is presented in Supplementary Figure 5.

To begin the comparative analysis of the two CD157 proteoforms, their respective cDNAs were cloned into a pCDNA3-based expression vector and stable transfectants established in HeLa cells (whose phenotype is CD157-negative/CD38-negative). Cell surface expression was assessed by flow cytometry using the SY/11B5 anti-human CD157 mAb. This mAb recognized membrane-bound CD157-001 and CD157-002 with similar mean fluorescence intensity (Fig. 3a), indicating epitope conservation in CD157-002.

The SY/11B5 mAb was used for western blot analysis of transfectant whole cell lysates. Canonical CD157 has four putative *N*-glycosylation sites and its molecular weight (MW) ranges between 42–50 kDa due to microheterogeneity of glycosylation patterns^36^ (Fig. 3b, left panel). CD157-002 also appears to be heavily and heterogeneously glycosylated (Fig. 3b, left panel). Western blotting was performed following deglycosylation with PNGase F, and again a visible difference in molecular weight (Fig. 3b, right panel) was evident between canonical CD157 and CD157-002, compatible with the presence of 15 additional amino acids (~1650 Da) in the latter. Although band intensities are similar but not equal in the glycosylated samples, there is a notable difference in intensity between the 001 and 002 deglycosylated bands, hinting at potential differences in binding efficiency in western blotting by the SY/11B5 mAb.

Next we wished to establish if there were differences in the subcellular localization of CD157-001 and CD157-002. Examination of HeLa/CD157-001 and HeLa/CD157-002 cell monolayers by confocal microscopy using SY/11B5 mAb showed overlapping expression patterns characterised by plasma membrane localization (Fig. 3c). These results indicate that, when expressed on the cell membrane, the two CD157 proteoforms have similar properties in SY/11B5 mAb binding and cellular distribution.

Finally, to bridge the gap between the HeLa model and a physiological situation in which both CD157-001 and 002 are co-expressed, we examined expression of CD157 in PMN by western blotting (Fig. 3d, left panel). The combination of low expression of CD157-002 in PMN coupled with the reduced ability of SY/11B5 to detect this proteoform when deglycosylated required the use of a polyclonal anti-CD157 antibody. The deglycosylated forms recognized by the polyclonal antibody showed comparable apparent molecular mass as the singularly expressed proteoforms in HeLa cells in Fig. 3b. The results obtained in PMN were corroborated in U937 myelomonocytic cells (Fig. 3d, right panel).

### CD157-002 is an efficient ECM receptor

**Figure 4:**
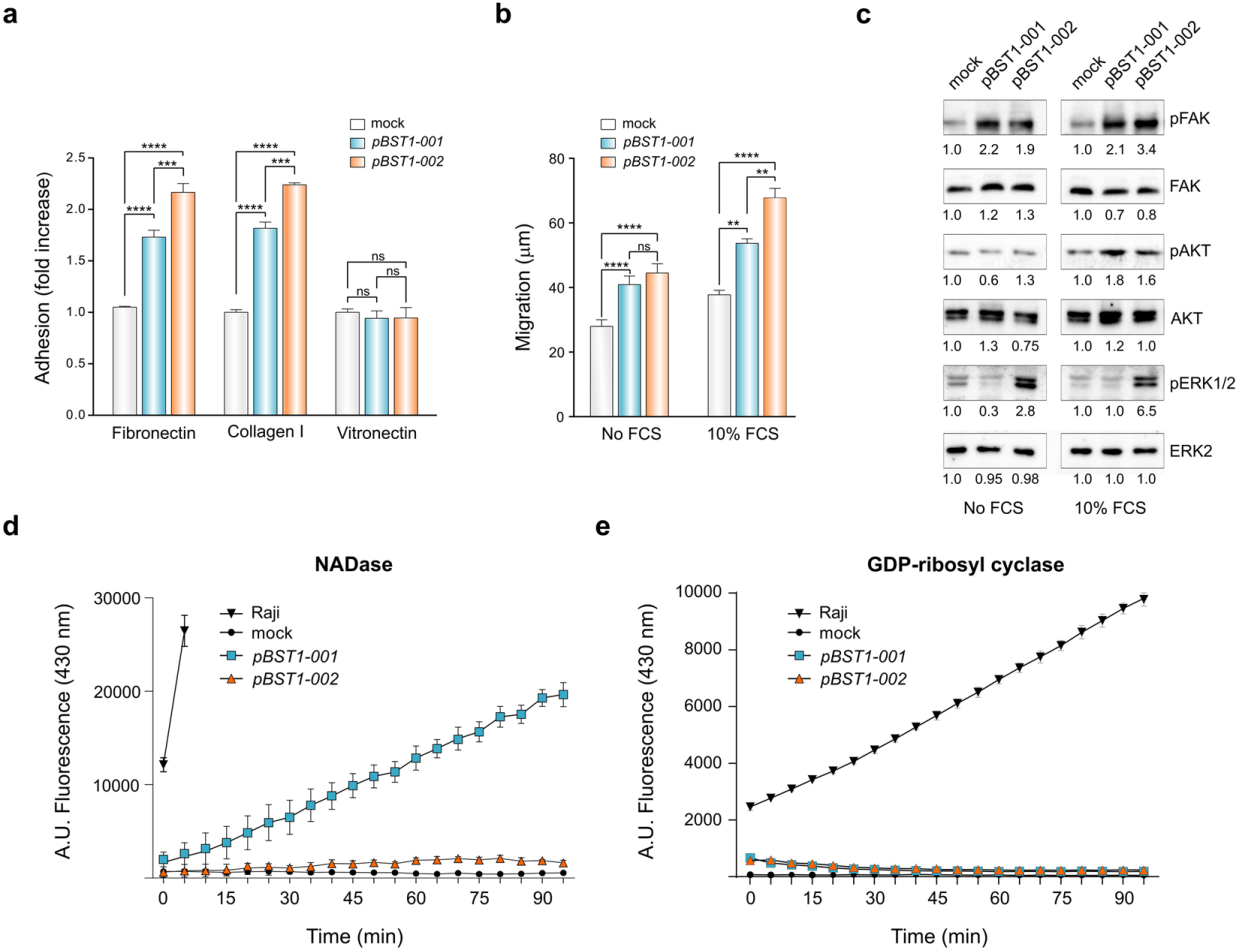
Comparative analyses of CD157 proteoforms in HeLa cell adhesion to ECM components, wound healing and enzymatic activities. (**a**) Histograms of adhesion assay with HeLa/*pBST1-001* (blue bars) and HeLa/*pBST1-002* (orange bars) transfectants show increase in adhesion to indicated substrates with respect to mock control (white bar). Cells were plated for 10 min at 37 °C onto fibronectin, collagen I or vitronectin. Results are expressed as fold-increase of cell adhesion in *BST1-001-* or *BST1-002*-transfected HeLa cells compared to mock-transfected cells, and represent the mean value ± s.e.m. of three experiments performed in sextuplicate. (**b**) Scratch-wound assay comparing the ability to migrate of HeLa/*pBST1-001* or *pBST1-002*. Results represent the distance in μm travelled by cells in 24 h determined as difference between the width of the wound at time 0 and at 24 h, and are expressed as mean ± s.e.m. of three independent experiments performed in triplicate. ****p < 0.0001; ***p < 0.001; **p < 0.01; *ns*, not significant; ANOVA with Dunnett’s multiple comparison test; **(c)** HeLa transfectants were incubated for 30 min without (left panel) or with FCS (right panel) then lysed. Total lysates (30 μg/lane) were resolved by 10% SDS-PAGE, immunoblotted and probed with the indicated antibodies. The numbers below each sample correspond to the relative protein density measured in HeLa/*pBST1-001* or *pBST1-002* compared with HeLa/mock and appropriate total protein. One representative experiment is shown (n = 3). Full-length blots are presented in Supplementary Figure 4 (lower panel). (**d**) Fluorimetric determination of ectoNADase activity or (**e**) ectoGDP-ribosyl cyclase activity in culture supernatants from HeLa/*pBST1-001* (blue squares), HeLa/*pBST1-002* (orange triangles) and HeLa mock transfectants (black circles) incubated with ε-NAD^+^ or NGD^+^, respectively. Raji Burkitt’s lymphoma cell line (CD38-positive, CD157-negative) was used as positive control. Accumulation of fluorescent products ε-ADPR or cGDPR was measured over time and is represented in arbitrary units (AU) of fluorescence intensity measured at 430 nm. Results are the mean ± s.d. of two independent experiments performed in triplicate.

As canonical CD157 is a well-characterised receptor that mediates leukocyte, mesothelial and specific tumor (EOC, MPM) cell adhesion to ECM proteins^6,11,15^, we conducted parallel studies to explore possible functional differences in the capacity of CD157-001 and CD157-002 to mediate cell attachment to fibronectin (FN), type I collagen (COL1) and vitronectin (VN) using the HeLa transfectant model. With respect to empty vector-transfected cells (designated as “mock”), expression of either CD157-001 or CD157-002 enhanced HeLa cell adhesion to microtiter plates coated with FN or COL1 (Fig. 4a). However, when comparing the two variants, HeLa/*pBST1-002* bound FN and COL1 more efficiently than HeLa/*pBST1-001*, a difference that was statistically significant (p < 0.001). As previously described for canonical CD157^11^, CD157-002 did not enhance adhesion to VN. Therefore, both CD157 proteoforms exhibit differential binding to discrete ECM proteins (*i*.*e*., to FN and COL1 but not to VN), with CD157-002 as efficient if not more so that CD157-001.

Wound healing assays were performed to compare the contribution of the two proteoforms to cell migration. In serum-free conditions, the migratory capacity of both CD157 proteoforms was similar, and greater than that of mock control (Fig. 4b). In the presence of 10% FCS, used as a source of ECM components, HeLa/CD157-002 again migrated more efficiently than cells expressing CD157-001 and this difference was statistically significant (p < 0.01).

We previously demonstrated that triggering CD157 by crosslinking with specific mAbs or adhesion to FN elicits phosphorylation of FAK (Tyr-397) leading to increased activation of the PI3K/AKT and MAPK/ERK1-2 pathways, and promotes actin cytoskeleton reorganization^6,11^. Therefore, activation of FAK and its downstream signalling effectors AKT serine/threonine kinase and ERK1/2 were compared in HeLa cells expressing either CD157-001 or CD157-002 maintained in basal culture conditions without FCS or following the addition of FCS as a source of ECM components. Western blotting showed increased pFAK Tyr-397 phosphorylation in both CD157 transfectants compared to HeLa mock control both in serum-free conditions and in the presence of FCS. The levels of total FAK were comparable in all cell lines. AKT Ser-493 phosphorylation was upregulated in both CD157 transfectants in the presence of FCS. Phosphorylated ERK1/2 was notably increased in CD157-002-expressing cells, both with and without serum, with comparable amounts of total ERK2 in all cell lines. Taken together, these results show activation of the FAK motility pathway associated with the expression of either CD157-001 or -002, and increased ERK1/2 activation in HeLa/*BST1-002* in line with the increased motility shown by these cells.

### Divergent enzymatic activity of canonical CD157 and CD157-002

CD157 has generated much less interest as an enzyme than its relatives CD38 and aplysia cyclase, possibly because CD157 appears to be a far less powerful catalyst than them^37^. However, there are at least two experimental models (involving homeostasis of hematopoietic and intestinal stem cells), albeit in mouse, that are reliant upon CD157-derived cyclic ADP ribose, even though it may be produced in hormone-like (*i*.*e*., subnanomolar) concentrations^23–25^. In the past, using sensitive HPLC methods, other labs demonstrated that human CD157 converted NAD^+^ to cyclic ADP ribose (minor product) and ADP ribose (major product), indicating the presence of ADP-ribosyl cyclase, NAD glycohydrolase and possibly cADPR hydrolase activities^19,20^. Limited experiments with analog substrates have detected similar activities in human CD157, using etheno-NAD^+^ (conversion products not quantified)^19^ or NGD^+^ (ca. 100 nmole/mg/min of cyclic GDPR produced)^38^.

Comparative analysis of CD157 enzymatic activity was carried out using whole HeLa cells expressing one or other of the two proteoforms. First, to test for NAD^+^ glycohydrolase activity, transfectants were incubated with the substrate 1,N (6)-etheno-NAD (ε-NAD^+^), a fluorescent analogue of β-NAD^+^. In the presence of canonical CD157, production of fluorescent etheno-ADP ribose (ε-ADPR) was observed, with the amount of product showing a linear increase over time (Fig. 4d). In contrast, when HeLa/CD157-002 cells were incubated with ε-NAD^+^, the fluorescence signal was negligible, indicating that CD157-002 does not share the NADase catalytic activity of canonical CD157. The NADase activity of the control Raji cell line, which strongly expresses CD38 but not CD157, was so high that after 10 min incubation ε-ADPR reached saturation and further measurement was not possible.

The nicotinamide guanine dinucleotide (NGD^+^) cyclization assay is frequently used as a proxy for ADP-ribosyl cyclase activity, with the caveat that these activities are not always equivalent^39^. HeLa cell transfectants and controls were incubated with NGD^+^ to measure GDP-ribosyl cyclase activity via production of cyclic GDP-ribose (cGDPR, a fluorescent analogue of cADPR). This assay was almost negative with both canonical CD157 and CD157-002. In contrast, incubation of Raji cells with NGD^+^ yielded easily detectable cGDPR accumulation in cell supernatants, consistent with the ability of CD38 to convert NGD^+^ mostly to cyclic GDP ribose (cGDPR) and little to GDPR^39^ (Fig. 4e). In summary, we detected a major difference in catalytic activity between CD157-001 and CD157-002.

### Conservation and dynamic evolution of *BST1* exon 1b in primate phylogeny

**Figure 5:**
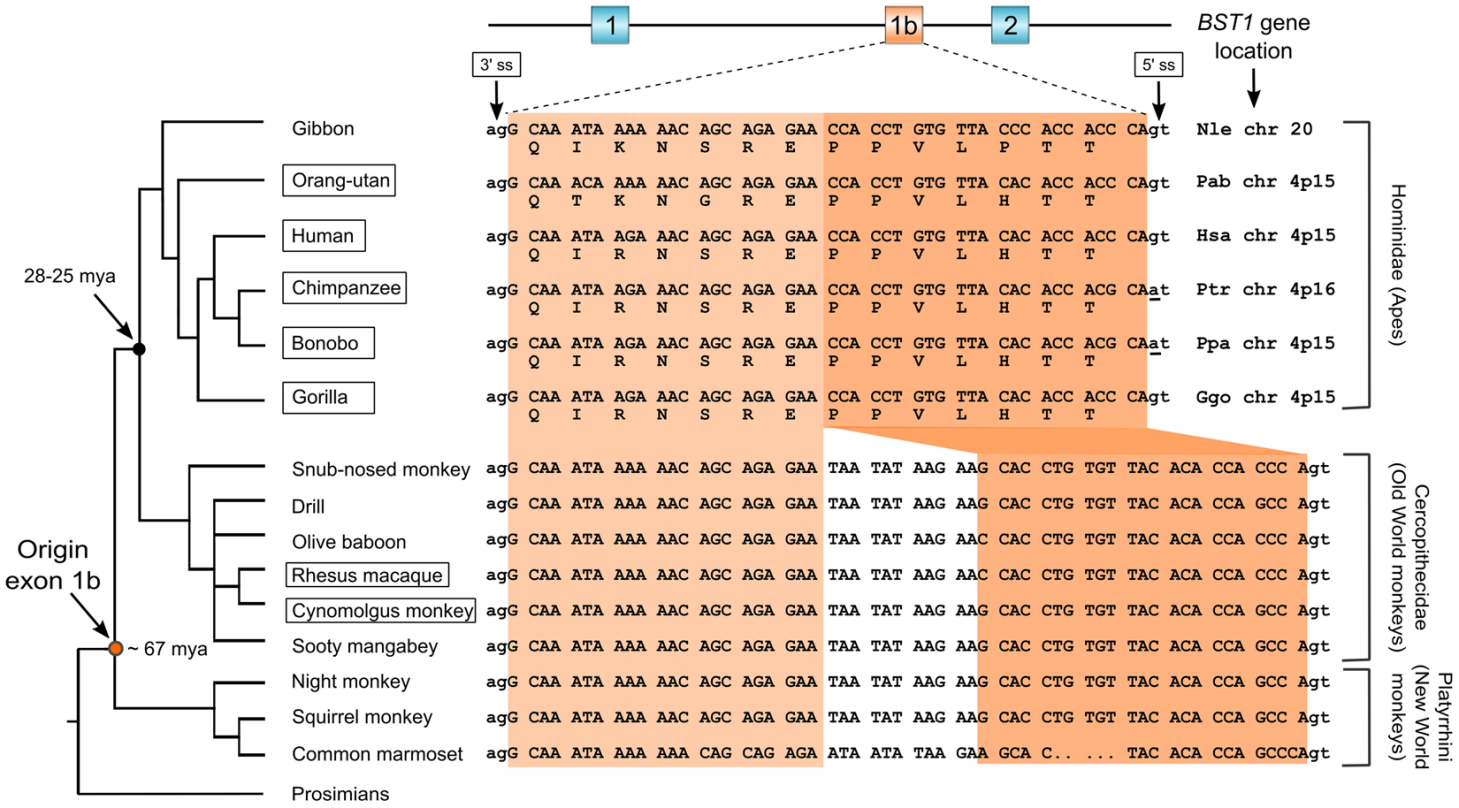
Origin, distribution and dynamic evolution of *BST1* exon 1b in primate phylogeny. Phylogenetic tree of primate species harboring exon 1b within the context of the *BST1* gene. Species are: gibbon (*Nomascus leucogenys*, Nle); orang-utan (*Pongo abelii*, Pab); human (*Homo sapiens*, Hsa); chimpanzee (*Pan troglodytes*, Pta); bonobo (*Pan paniscus*, Ppa); gorilla (Gorilla gorilla, Ggo); snub-nosed monkey (*Rhinopithecus roxellana*); drill (*Mandrillus leucophaeus*); olive baboon (*Papio anubis*); rhesus macaque (*Macaca mulatta*); cynomolgus monkey (*Macaca fascicularis*); sooty mangabey (*Cercocebus atys*); night monkey (*Aotus nancymaae*); squirrel monkey (*Saimiri boliviensis*); common marmoset (*Callithrix jacchus*); prosimians (lemur, loris, tarsier). Alignment of the exon 1b sequence is shown, highlighting the correlation between exon sequence evolution and phylogenetic lineage. Species with boxed names have genome sequences that are published or in progress. Chimpanzee (*Pan troglodytes*) and bonobo (*Pan paniscus*), part of the *Pan* genus lineage, both show AT mutation at the exon 1b 5′ splice site (5′ ss). An 11 bp sequence of exon 1b is deleted in the ape lineage after its separation from Old World monkeys approximately 28–25 mya^52^. Exon 1b origin is estimated to have occurred after the anthropoid/tarsier separation approx. 67 mya (orange dot).

The 10-exon structure of *BST1* in the human is a departure from the conserved 9-exon structure of the gene found in other mammalian (mouse, rat) and non-mammalian (chicken) species (EF, unpublished result). To establish if the 10-exon structure of the *BST1* gene represents a human innovation, the evolutionary history of exon 1b was reconstructed by database BLAST analyses which retrieved homologous nucleotide sequences in 11 species, limited to primates (Fig. 5). In all of these species, the exon 1b-like sequence was found within the *BST1* gene and always in orthologous positions between exons 1 and 2. Exon 1b-like sequences were not found in tarsier (*T*. *syrichta*), lemuriforms (*Microcebus*) nor Rodentia (mouse, rat), suggesting that this exon was acquired early in primate phylogeny after the separation of simians from tarsiers, estimated to have occurred approximately 67 million years ago (mya) (http://timetree.org).

Visual inspection of the alignment shows that although highly conserved, the exon 1b sequence has evolved over time, and apes have a shorter exon than monkeys because of an 11 bp deletion (Fig. 5). Among apes, whose genomes-to-proteomes databases are more complete than most of the monkey databases, there is evidence that not only human but also gorilla (*Gorilla gorilla*) and orangutans (*Pongo abelii*, *Pongo pygmaeus*) express both constitutive 10-exon and alternatively spliced 9-exon transcripts. In gibbon (*Nomascus leucogenys*), chimpanzee (*Pan troglodytes*) and bonobo (*Pan paniscus*), we have currently found only 9-exon-derived transcripts and proteins. However, in chimp and bonobo, closer nucleotide sequence examination revealed that the consensus GT donor splice site is mutated to a nonconsensus AT donor splice sequence (5′ splice sites, Fig. 5), possibly precluding use of exon 1b in chimp and bonobo. As both these species belong to the genus *Pan*, the G → A substitution may represent a lineage-specific modification. The impact of exon 1b on CD157 in Old and New World monkeys is a work in progress as the relative databases are currently less informative, although there is some preliminary evidence in macaques of a different splicing pattern of exon 1b.

## Discussion

The outcome of this study has gone beyond the expected results, leading us to: (i) redefine the structure of the human *BST1* gene following the inclusion of a previously unidentified exon; (ii) identify a new *BST1* transcript firstly in human neutrophils and then in other tissues, and demonstrate expression of both proteoforms in PMN; (iii) characterise the divergent functional characteristics of the novel proteoform CD157-002 with respect to the canonical CD157-001 form; (iv) reveal that canonical CD157-001 is generated by alternative splicing; and (v) trace the origins of the new exon 1b within the primate phylogenetic tree.

Firstly, a static situation has changed. The human *BST1* gene and cDNA were characterised in the 1990’s using standard molecular cloning procedures. The resulting 9-exon organization of the human gene was structurally homologous to the murine counterpart *bst1/bp3*. In addition, homologous cDNAs, cloned from mouse and rat, were available so there was no reason to suspect that the human cDNA was anything other than the product of constitutive splicing whereby all of a gene’s exons are joined together in the order in which they occur in the genome. This static image of *BST1* has now started to change, thanks to the chance discovery of a novel *BST1* exon found by experiment, and supported by abundant genomic, transcriptomic and proteomic data available *in silico*.

The process of joining different combinations of exons by AS is a powerful means of increasing protein diversity^40,41^, and is calculated to occur, at least at the transcriptional if not at the protein level^42^, in >90% of human genes^41^. Among the ARCs, human CD157 is the first family member to be described as being regulated by AS. What is intriguing about AS in CD157 is that it is not used to add to phenotypic diversity but, on the contrary, to maintain the status quo. By skipping exon 1b, the canonical proteoform of CD157 maintains the 1:1 sequence conservation and functional characteristics of its orthologues in mouse (NCBI accession number Q64277), rat (Q63072) and chicken (XP_420775). This seems to conform with observations that have emerged from large-scale proteomics experiments indicating that, despite AS being so widespread, most protein-coding genes have one dominant proteoform^43,44^.

How did a novel *BST1* exon arise during primate evolution? We can only speculate on the origins of *BST1* exon 1b, which may have occurred following exonization of an intronic sequence, an event estimated to occur at the rate of 2.71 × 10^−3^/gene/million years^45^. By comparative phylogenomics, exon 1b orthologous sequences were not detected in other mammals apart from within the primate suborder Anthropoidea (monkeys and apes including human), suggesting that the birth of exon 1b may have occurred after the separation of the anthropoids from their sister group, the tarsiers, approximately 67 mya. Newly born exons may evolve rapidly and disappear if unconstrained by function^46,47^. So far, exon 1b nucleotide sequences located in orthologous positions within the *BST1* gene have been found in all simian genomes available for us to examine, suggesting that selective constraints have prevented exon 1b from disappearing without trace. In addition, it is clear exon 1b is a dynamically evolving exon: with respect to monkey, it has undergone an 11 bp deletion in apes where the peptide insert is certainly included to generate longer forms of CD157, as shown here. Transcriptional and proteomic information in most Old and New World monkeys is currently lacking, but data from the more detailed macaque genomes seem to suggest that the exon is spliced and incorporated into novel proteoforms (*work in progress*).

In human *BST1*, inclusion of exon 1b (albeit at low level) into a transcript by constitutive splicing has the potential to add to the phenotypic variability of CD157. The impact of the inclusion of the 15 aa sequence into the CD157 polypeptide does not appear to be catastrophic for overall 3D protein structure, at least as judged by correct intracellular trafficking and localization on the cell membrane with recognition by a specific monoclonal antibody, in addition to the unaltered, or indeed enhanced, ability to act as an adhesion protein and signalling molecule. However, the deleterious effect of exon 1b insertion on CD157-mediated NADase activity appears to be remarkable and probably explains the use of AS as a means of maintaining the catalytically active form of CD157. Examination of the 3D model presented here shows that the exon 1b-encoded 15 aa insert is predicted to form a structurally disordered, hydrophilic loop protruding from the membrane-distal surface of the protein. Downstream of the insertion, the CD157-002 polypeptide structure prediction appears to overlap with the 3D structure of CD157-001. On the one hand, this conservation of structure would not be expected to lead to changes in ECM component binding. On the other hand, the model does not offer an immediate explanation for the loss of the NADase activity in CD157-002. It is not obvious how the loop could hinder substrate entry or binding into the catalytic cleft, but further investigation into the capacity of CD157-002 to bind NAD^+^ is warranted. It is also not clear what structural and functional effects the insert could have on the first N-glycosylation site, located three amino acids downstream of the insertion.

The use of AS to maintain a catalytically active form of CD157 raises the question why maintain a catalytically inactive variant? Among the possibilities, CD157-002 may create new protein-protein interactions, as suggested by recent findings that protein isoform pairs share less than 50% of their interactions^48^. Alternatively, CD157-002 may be a more effective ECM receptor, a possibility not excluded by the experimental evidence presented here, without the NAD-consuming activity which may impact upon NAD^+^ homeostasis^49^. Last but not least, CD157-002 may possess additional functional characteristics that were undetectable with the assays performed here. Whatever the distinctive functional properties of CD157-002, the fact remains that, so far, the two CD157 proteoforms always seem to be co-expressed. Therefore, when performing functional experiments that evaluate the role of CD157 in physiological or pathological conditions, the outcome will be determined by the relative contribution of the two proteoforms.

In conclusion, *BST1* is proving to be a prime example of a gene that has deployed all of the major mechanisms that increase phenotypic diversity, from its origin by tandem gene duplication and diversification, to multifunctionality to AS with inclusion/skipping of a novel cassette exon. Our findings add a new level of complexity to all the analyses in which CD157 is being evaluated for its relevance to human disease or its suitability as a therapeutic target in selected diseases^16^. Perhaps sibling rivalry between CD157 and CD38 has just begun.

## Methods

### Antibodies, cell lines and reagents

Anti-CD157 mAb (SY/11B5, IgG1) was produced in-house and affinity purified on protein G. The sheep anti-human CD157 polyclonal antibody (AF4736, R&D Systems). Horseradish peroxidase (HRP)-conjugated anti-mouse IgG and anti-sheep IgG, and anti-β-actin-HRP were from Santa Cruz Biotechnology. Alexa Fluor-488-F(ab’)_2_-GαMIgG was from Invitrogen (Life Technologies). FITC-labelled F(ab’)_2_ RαMIgG [F(ab’)_2_-RαMIgG-FITC] was from Jackson ImmunoResearch.

Human cell lines THP1 and U937 (myelomonocytic leukaemias) and HeLa (cervical cancer) were originally obtained from the American Type Culture Collection. Cells were maintained at 37 °C in RPMI 1640 with 2.5 μg/ml amphotericin B, 100-unit penicillin, 100 μg/ml streptomycin and 10% FCS. Cell cultures were routinely checked for the absence of mycoplasma contamination by PCR. Fibronectin, collagen I, vitronectin, crystal violet, propidium iodide, 1,N^6^-ethenoadenine dinucleotide (ε-NAD^+^) and nicotinamide guanine dinucleotide (NGD^+^) were from Sigma Aldrich.

### Isolation of human PMN

PMN were isolated to ≥95% purity from peripheral blood of healthy volunteers, as described^5^. All participants gave written informed consent. All experiments were carried out in accordance with relevant guidelines and regulations, approved by the Institutional Review Board (Department of Medical Sciences, University of Torino).

### Analysis of gene expression by RT-PCR

Total RNA was extracted from 2 × 10^6^ PMN collected in TRIzol® reagent (Invitrogen) using Direct-Zol^TM^ RNA MiniPrep Kit (Zymo Research) according to the manufacturer’s instructions. Total RNA tissue samples were purchased from Invitrogen. Using oligo-dT primers, 2 μg RNA were reverse transcribed with M-MLV Reverse Transcriptase (ThermoFisher Scientific), according to the manufacturer’s instructions. cDNA from selected cell types and tissues were from Human MTC™ Panel II (Clontech Laboratories). The cDNAs were amplified (30 cycles) using KAPA2G Fast HotStart DNA polymerase (Kapa Biosystems). Each cycle consisted of denaturation at 94 °C for 10 s, annealing for 10 s and extension at 72 °C for 1 s, followed by a final extension at 72 °C for 40 s. For co-amplification of *BST1-001* and *-002* transcripts, primers were: Ex1/F 5′-ACACTTGCGGGACATCTTCC-3′; Ex2/R 5′-GGGAATAGAGTGCCTGGACA-3′. For *BST1-002*-specific amplification, primers were: Ex1b/F 5′-GCAGAGAACCACCTGTGTTA-3′; Ex2/R 5′GAACATCGCTCAGGGGCATA-3′. Annealing temperature (Ta°) was 57 °C for both primer pairs. β-actin primers were: forward 5′-ATGGATGATGATATCGCCGCG-3′; reverse 5′-CTAGAAGCATTTGCGGTGGACGATGGAG-3′. Ta° was 55 °C. Negative control reactions with only a single primer, with RNA without reverse transcription, or in the presence of RNase were performed. PCR products were analysed by agarose gel electrophoresis and visualized by staining with Midori Green (Nippon Genetics).

### PCR product sequencing

PCR products were purified of unused primers and nucleotides by treatment with ExoSAP-IT® (Amersham-Pharmacia Biotech), followed by direct sequencing using the ABI PRISM® BigDye^TM^ Terminator Cycle Sequencing kit (Applied Biosystems) and analysis on an ABI 3130 Genetic Analyzer with Sequencing Analysis software (Applied Biosystems).

### Real-Time Quantitative PCR expression analysis

Real-Time quantitative RT-PCR (RT-qPCR) was carried out using an ABI_Prism 7500 Fast System (Applied Biosystems) with TaqMan Gene Expression Master Mix (Applied Biosystems) and Universal Probe Library (UPL) technology (Roche) according to the manufacturer’s instructions. Primers and UPL probes used for RT-qPCR were: for *Bst1-001* transcript, forward 5′-CACACTTGCGGGACATCTT-3′ and reverse 5′-TGTCCAGGCACTCTATTCCC-3′, UPL no. 76; for Bst1-002 transcript, forward 5′-CAGCAGAGAACCACCTGTGTTA-3′; reverse 5′-TGTCCAGGCACTCTATTCCC-3′, and UPL no. 76. Each sample was examined in triplicate, in three independent experiments. Experimental Ct values were normalized to the housekeeping gene *GUSB*, used as endogenous control (VIC-labelled predesigned TaqMan gene expression assays, GUSB, 4326320E, Applied Biosystems). The relative quantity of each transcript was calculated following normalization to *GUSB* using the conventional comparative (ΔΔCt) method.

### Constructs and cell transfection

Plasmid constructs directing expression of canonical CD157 or CD157-002 were prepared by cloning the relative human full-length cDNAs into pCDNA3-puro, an in-house modified variant of the pCDNA3 eukaryotic expression vector (Invitrogen) that contains a puromycin resistance (*puro* ^*r*^) cassette. Plasmids were transfected into HeLa cells using Nanofectamin (PAA) according to the manufacturer’s protocol. HeLa cells transfected with empty pCDNA3 vector (indicated as mock) were used as controls. Stable transfectants were selected for 3 days in medium with 5 µg/ml puromycin (SantaCruz Biotechnology) yielding ~100% transfected cells.

### Flow cytometry and confocal microscopy

For flow cytometric analysis, cells (2 × 10^5^) were incubated with SY/11B5 mAb (5 μg/ml) for 30 min at 4 °C followed by F(ab’)2-GαMIgG-FITC for 30 min at 4 °C. Fluorescence was analysed by means of a FACSCanto flow cytometer using CellQuest software (BD Biosciences). Background mAb binding was estimated by means of isotype-matched control IgG. Three independent readings were obtained from separate experiments. For confocal microscopy, cells were grown on glass coverslips for 24 h, washed with HBSS and fixed with 2% paraformaldehyde (PFA). Coverslips were then blocked with 2% goat serum in HBSS for 30 min at RT, before incubation with anti-CD157 mAb and Alexa Fluor-488-F(ab’)2-GαMIgG, and staining with propidium iodide. Samples were analysed using an Olympus FV300 laser scanning confocal microscope and FluoView 300 software (Olympus Biosystems). Cells were imaged using a ×60 oil immersion objective (1.4 NA).

### Cell adhesion assay

HeLa cell transfectants (3 × 10^4^ in serum-free RPMI 1640) were plated in 96-well plates, previously coated with 10 μg/ml fibronectin, collagen 1 or vitronectin and blocked with 0.2% BSA. After 10 min adhesion at 37 °C, nonadherent cells were removed by washing. Adherent cells were fixed with 2% PFA and stained with crystal violet solution (0.5% wt/vol in H_2_O) for 20 min, washed and solubilized in triton X-100 (1.0% solution in H_2_O). The absorbance readings were recorded at 595 nm using a microplate reader (Infinite® 200 PRO). The absorbance of HeLa cell transfectants were compared with those of mock cells (basal HeLa cell adhesion) and expressed as fold increase of cell adhesion.

### Wound healing assay

Cells were seeded in 24-well plates until confluence and a scratch was then made across the monolayer with a sterile pipette tip. The remaining monolayers were then gently rinsed with serum-free medium to remove detached cells and debris, and serum-free medium or medium with 10% FCS was added, as indicated. Wounds were photographed at the beginning of the experiment (t = 0) and at 24 h, and at least 20 randomly chosen distances across the wound for each time point were measured and the mean calculated. The distance travelled by the cells was determined as the difference between the width of the cell-free area after 24 h and at time 0. Wound images were acquired using an Olympus Biosystems Microscope IX70, equipped with a UC-30 camera and CellF software (Olympus Biosystems).

### Western blotting and phosphorylation assay

Total lysates of HeLa cell transfectants, PMN and U937 cells were obtained in ice-cold RIPA buffer supplemented with protease inhibitor cocktail (Sigma Aldrich). Where indicated, cell lysates were treated with N-glycosidase (PNGase F) (Sigma Aldrich) as previously described^11^. Protein extracts (30 μg/lane) were separated by: for Hela cells, 12% SDS-PAGE under non-reducing conditions and transferred to PVDF membranes (Millipore); for PMN and U937, by 4-12% gradient SDS-PAGE under reducing conditions and transferred to nitrocellulose membranes. After blocking with 5% milk, membranes were probed with anti-CD157 mAb or anti-CD157 polyclonal antibody followed by the appropriate HRP-conjugated anti-IgG, or with anti-β-actin-HRP. Immunoreactive bands were visualized by Westar ECL substrate (Cyanagen). For phosphorylation assays, HeLa cell transfectants were serum-starved overnight, and then incubated for 30 min at 37 °C in fresh medium without or with 10% FCS. Cells were then placed on ice, treated with 1 mM pervanadate and lysed with RIPA buffer supplemented with 1 mM Na_3_VO_4_, 5 mM NaF, 50 μg/ml aprotinin and leupeptin. Lysates were run on 10% SDS-PAGE under reducing conditions and transferred to PVDF membranes. Membranes were blocked (2% BSA) and probed with anti-phospho-FAK (Tyr-397), anti-phospho-AKT (Ser-493) and anti-phospho-ERK1/2 (Tyr-204/Tyr-187) (Santa Cruz Biotechnology), then stripped and reprobed with anti-FAK, anti-AKT and anti-ERK2 antibodies (Santa Cruz) followed by incubation with the appropriate HRP-conjugated antibodies. Immunoreactive bands were visualized by ECL. Images were acquired with a ChemiDoc XRS+ System, and densitometric analysis was performed using Image Lab v5.2.1 software (Bio-Rad). Quantification of protein band density was performed for each individual protein according to the formula:

$$\frac{Phospho-protein\,density\,of\,HeLa\,transfectants/phospho-protein\,density\,of\,HeLamock}{Total\,protein\,density\,of\,HeLa\,transfectants/total\,protein\,densityof\,HeLa\,mock}$$

### NAD^+^ glycohydrolase and NGD^+^ cyclase enzymatic activities

HeLa cell transfectants cells were detached in 2 mM EDTA, centrifuged 1 min at 4,000 rpm, suspended at 4 × 10^5^ in 10 mM potassium phosphate buffer, pH 7.4, and transferred to 96-well optical bottom black plates (NUNC) at 1 × 10^5^ cells/250 μl/well, in triplicate. Substrate was added to intact cells and the reaction was measured over 95 min at 37 °C. Ecto-NAD glycohydrolase activity was measured continuously by fluorescence-based assay using 40 μM ε-NAD^+^ as substrate. Cyclase activity was evaluated by cyclization of the NAD^+^ surrogate substrate NGD^+^ to its fluorescent derivative cyclic GDP ribose (cGDPR) whose production was measured continuously in the presence of 100 µM NGD^+^ as substrate. Hydrolysis of ε-NAD^+^ and cyclization of NGD^+^ were determined by measuring the accumulation of fluorescent reaction products over time using an Infinite® F200 (Tecan) fluorescence plate reader with excitation and emission wavelengths of 300 nm (BW 20 nm) and 430 nm (BW 35 nm), respectively. The rate of ε-ADPR or cGDPR production was determined from the slope of fluorescence increase. Data are reported in arbitrary units (A.U.) of fluorescence intensity at 430 nm.

### Bioinformatics

To identify homologous sequences, the human *BST1* exon 1b nucleotide and protein sequences were used to query genomic databases by BLASTN and BLASTP searches, respectively. In most cases, retrieved sequences were annotated with information on their placement within a gene and chromosomal location. The exon 1b homologs included here were all located in orthologous positions within the BST1 gene of the species in question. Sequence alignments was obtained with MAFFT version7 (http://mafft.cbrc.jp/alignment/server/). Protein structure homology modelling was performed using the SWISS- MODEL program using PDB structure 1ISI of CD157 complexed with etheno-NAD^+^^35^. The primate species tree was generated using the Common Tree tool at https://www.ncbi.nlm.nih.gov/taxonomy. Databases used for multispecies analysis of the *BST1* gene and proteins were: (NCBI https://www.ncbi.nlm.nih.gov/), Ensembl (www.ensembl.org/) and UCSC (https://genome.ucsc.edu/) genome browsers. Intrinsic disorder in the human CD157 protein was predicted using IUPred (http://iupred.enzim.hu/)^34^. Putative branch points were predicted using Human Splicing Finder (http://www.umd.be/HSF/), version 2.4.1.^50^ and Sroogle (http://sroogle.tau.ac.il/)^51^.

### Statistical methods and data analysis

Unless otherwise indicated, results are expressed as means ± sem. Comparisons between two groups were carried out using the Student’s t test. One-way analysis of variance (ANOVA) with Dunnett’s post hoc analysis was used for multiple comparisons. Statistical analyses were performed using GraphPad Prism 7 software (San Diego, CA). For all analyses, differences were considered significant at p < 0.05 (*p < 0.05, **p < 0.01, ***p < 0.001, ****p < 0.0001 versus control; ns, not significant).

All data generated or analysed during this study are included in this published article (and its Supplementary Information files).

## Electronic supplementary material

Supplementary Information
