# Supplementary material for: Human canonical CD157/Bst1 is an alternatively spliced isoform masking a previously unidentified primate-specific exon included in a novel transcript
Source: Sci Rep. 2017 Nov 21;7:15923. doi: 10.1038/s41598-017-16184-w (PMC5698419; doi:10.1038/s41598-017-16184-w)
Supplement: Supplementary file 1 — Supplementary Information [file 41598_2017_16184_MOESM1_ESM.pdf]

Human canonical CD157/Bst1 is an alternatively spliced isoform masking a previously unidentified primate-specific exon included in a novel transcript

Enza Ferrero[1*], Nicola Lo Buono[1,2], Simona Morone[1], Rossella Parrotta[1], Cecilia Mancini[3], Alfredo Brusco[3], Alice Giacomino[1], Stefania Augeri[1], Antonio Rosal-Vela[4], Sonia García-Rodríguez[4], Mercedes Zubiaur[4], Jaime Sancho[4], Alessandra Fiorio Pla[5] and Ada Funaro[1*]

[1]Laboratory of Immunogenetics, Department of Medical Sciences, University of Torino, 10126 Torino, Italy.

[2]San Raffaele Diabetes Research Institute, San Raffaele Hospital, 20132 Milano, Italy.

[3]Laboratory of Medical Genetics, Department of Medical Sciences, University of Torino, 10126 Torino, Italy.

[4]Department of Cellular Biology and Immunology, Instituto de Parasitología y Biomedicina López-Neyra, IPBLN-CSIC, Parque Tecnológico de la Salud de Granada, 18016 Granada, Spain.

[5]Department of Life Sciences and Systems Biology, University of Torino, 10123 Torino, Italy.

*Corresponding authors

Enza Ferrero: enza.ferrero@unito.it

Ada Funaro: ada.funaro@unito.it

SUPPLEMENTARY INFORMATION

Figure legends

Supplementary Figure 1. Genomic nucleotide sequence of human *BST1* from exon 1 to exon 2, encompassing exon 1b. Nucleotide sequence of human *BST1* from the 5’ end of exon 1 to the 3’ end of exon 2. Exons 1 and 2 are shaded in blue; exon 1b is shaded in orange. GT/AG splice sites are shaded in yellow. The branch point (open box) and polypyrimidine tract (grey box) associated with exon 1b are indicated. The nucleotide sequence was analysed for splicing signals using Human Splicing Finder[1] and the SROOGLE program[2].

Supplementary Figure 2. Quantification of *BST1* transcripts. Expression levels of *BST1-001* (top panel) and *BST1-002* (lower panel) in ten PMN samples purified from peripheral blood of healthy subjects. Transcript expression was calculated relative to the *GUSB* endogenous control, using the ΔΔCt method. Bars represent the fold change of each sample relative to the mean of all samples analysed. Each PMN sample was examined in triplicate (n = 3).

Supplementary Figure 3. Uncut gels for *BST1* expression profiles by RT-PCR.

Supplementary Figure 4. Original gels for western blotting. Top panel show gels from which band images were obtained for CD157 detection by western blotting; bottom panel shows original gel strips for phosphorylation analyses by western blot.

Supplementary Figure 5. Original blot showing CD157 in PMN and U937.

Supplementary References

1. Desmet, F. O. *et al.* Human Splicing Finder: an online bioinformatics tool to predict splicing signals. *Nucleic acids research* **37**, e67, doi:10.1093/nar/gkp215 (2009).
2. Schwartz, S., Hall, E. & Ast, G. SROOGLE: webserver for integrative, user-friendly visualization of splicing signals. *Nucleic acids research* **37**, W189-192, doi:10.1093/nar/gkp320 (2009).

Exon 1

```
   1 GAGAAGGGGA GTGGAGGAAG CACGGGACTG GAGGGACCAA AGTTCCCCGA TGGCGGCCCA
  61 GGGGTGCGCG GCATCGCGGC TGCTCCAGCT GCTGCTGCAG CTTCTGCTTC TACTGTTGCT
 121 GCTGGCGGCG GGCGGGGCGC GCGCGCGGTG GCGCGGGGAG GGCACCAGCG CACACTTGCG
 181 GGACATCTTC CTGGGCCGCT GCGCCGAGTA CCGCGCACTG CTGAGTCCCG AGCAGCGGTG
 241 AGGCAGTCGG CCGGGTGGAA GGGGAGCCGG AAAGAGGCAA CGGTGGGGAG GGCCTGGGGA
 301 GGGGAAAACT GGCGCTAAAG TTCGGGGTGA GGGGGCAATG AGAGAGGCCT TGAGGGGAGA
 361 GGTGAGTGTG GAGACAGCGA TGGTCCTGAA CGATGGGGGC GAGTGAGGAG GGGGCTGTGA
 421 AGTGTGTCTG CAGGTGAGGG GTGCGCTGTG CGGGGTCTAG AGATGAGTGT GTGTGTGTGC
 481 GCGCACACAC ACACTGTAGA GGTGAGGGGT GTGTGCGTGT GTTCTAGAGG TGAGGGGGGT
 541 GTGTGTGTGC GCGCGCTCTA GAGGTGGGAG GTGTGTGTGT GCTCTAGAGG TGGATGTGTG
 601 TGTGTGTGCT CTAGAGGTGG GGGTGTGTGT GTGTGTGTGT GCTCTAGAGG TGAGGGGTGT
 661 GTGTGTGTGT TCTAAAGGTG AGATGTGTGT GTGTGTTCTA GAGGTAAGGG GTGTGTGTGT
 721 TCCAGAGGTG AGGTGTCTGT ATGTGTGTTC TAGAGGTGAG GGGTGTGTGC ATGTGTGTTC
 781 TAGAGGTGAA GGGGGTGTGT GTGCGTGCGC TCTAGAGGTG AGGGGTGTGT GTATTCTAGA
 841 GGTGAGGGGT GTGTGTGTTC TAGAAGTGAG GGGTGTGTGT GTTCTGGAGG TGAGATGTGT
 901 GTGTGTGTGT TCTAGAGGTG AGGGGTGCGT GCATGTGTTC TAGAGGTGTG TGTGTATCTG
 961 TGTTCTAGAG GTGAGGGGTG TGTGTGTGTA GTCTAGAGGT GACAGGTGTG TGTGTGTGGT
1021 CTATAGGTGA GGGATGTGTG TGTGGTCTAG AGGTGAGGGA TGTGTGTGTG GTCTAGAGGT
1081 GAGGGGTGTG TGTGTTCTAG AGGTGAGGGG GTGTATGTGT ATTCTAGAGC TGAGGAGAGT
1141 GTGTGTTCTA GAGATGAGGT GTGTATGTGT GTTCTAGAGG TGAGGGGTGT GTGTGTGTTC
1201 TGGAGTTGAG GGGTGTGTGT TCTAGAGGTG AGATGTGTGT GTTCTAGAGA TGATGGGTGT
1261 GTGTGTTCTA GAAGTGAGAG GTGTGTGTGT TCTAGAGGTA AGGGATGTGT GTGTGTGTGT
1321 GTTCTAGAGG TGAGTTGTGT GTGTGTTCTA GAGGTGAGAT GTGTGTGTGT TTGTTCTAGA
1381 GGTGAGGGGT GTGTGTGTGT AGTCTAGAGG TGACAGGTGT GTGTGTGTGG TCTATAGGTG
1441 AGGGATGTGT GTGTGGTCTA GAGGTGAGGG ATGTGTGTGT GGTCTAGAGG TGAGAGGTGT
1501 GTGTGAGTGT GAAGGAGGGT GGAGAGGTAT CCCTCTAGAA GTGAGGGTGG GGTAGAGGTG
1561 AACTGGGTAA CAGCCCATTG CCACCCAGCA GATGTTTAGT CTGAATACTC AGAACCCAGA
1621 ATGTGATCTC AGGTTGGAAT TCCTTGAGTC CAGTCCTTCC AGAATTCAAA TTTCAGATCC
1681 CCTAAATCTG GAGGATTTCT GAAGTTTGGG TCTTTCTTGC CAGTGCTGCA TTTCTTACTG
1741 ATTTTTGTTC CTTCTGTGGC CCCCCTTTTC TTATGTTTGT GATGTCTTTC TTGTGCTGAT  <- Branch point
1801 CATTTTTATT GTCGCACATT TCAGGCAAAT AAGAAACAGC AGAGAACCAC CTGTGTTACA
1861 CACCACCCAG TATGAGAAAC AAAGTTACAA ATGCTATGGG AAATATTTTT TGAAAACTAG
1921 CATGGACCTC AAGAGGGGCT TGCACTGGAG GGTGCACCTG CTTTGCCAGA GGTGGAAGCC
1981 TTTTGCAGCA TTGGGCCTCC CTCCTAACAC TACACATCCT GCTGGCTGGC CTGGCCTCAC
2041 CTGGTCCCCG AGCTCCACAC TGGAACTGGA CTATGAGACT GGCCCCACCG TTAGGTCTAA
2101 ATGAAGAGGC ACCTGCTGTA TAGTTGACTG AATTTGTCAC CTCCTGCTCG GTGCCATTTC
2161 TGCTTGTCTC CGTGAGCCTG GGGCAAGGCT GTATTCCTTA GCTCTGAGAA ATCTGTGCAG
2221 TGCCTTCAGA GAGACAATTC CAGTGTCAGA TTTCTGAATC TGTCACCTAA GCCATGACAA
2281 TTCGTTGTCT TTTTTGTAAG CCTACTTCTG CAGTTACTAT ATGAAAAATC TCTTGTAAAG
2341 CTCTTAGACA AATGGGCATA CTTCACAACA CATTATGATG TGCATGTGTG TGTTCTTCCC
2401 AACTTGTACT TTTGCACAGG AACAAGAACT GCACAGCCAT CTGGGAAGCC TTTAAAGTGG
2461 CGCTGGACAA GGATCCCTGC TCCGTGCTGC CCTCAGACTA TGACCTTTTT ATTAACTTGT
2521 CCAGGCACTC TATTCCCAGA GATAAG
```

Polypyrimidine Tract

Exon 1b

Exon 2

Branch point

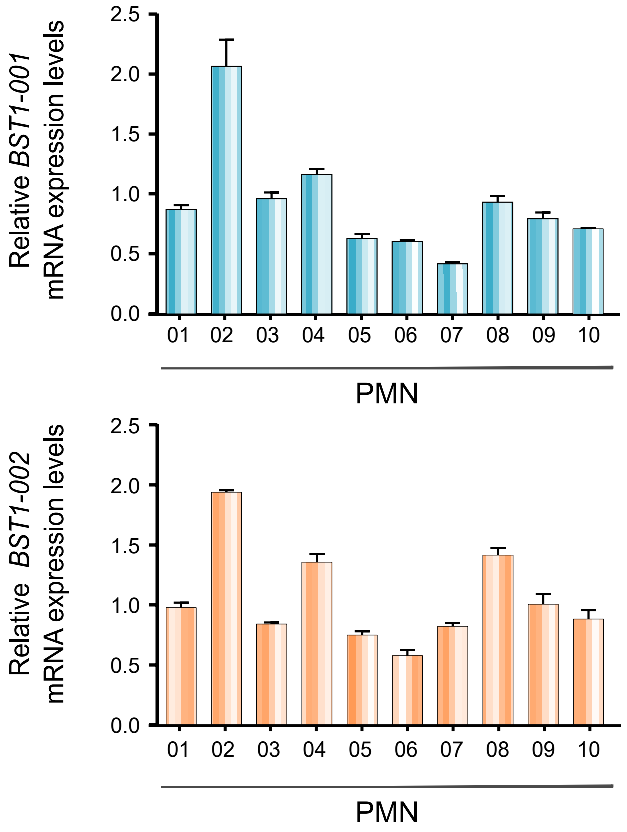
Relative BST1-001 mRNA expression levels
2.5
2.0
1.5
1.0
0.5
0.0
01 02 03 04 05 06 07 08 09 10
PMN
Relative BST1-002 mRNA expression levels
2.5
2.0
1.5
1.0
0.5
0.0
01 02 03 04 05 06 07 08 09 10
PMN

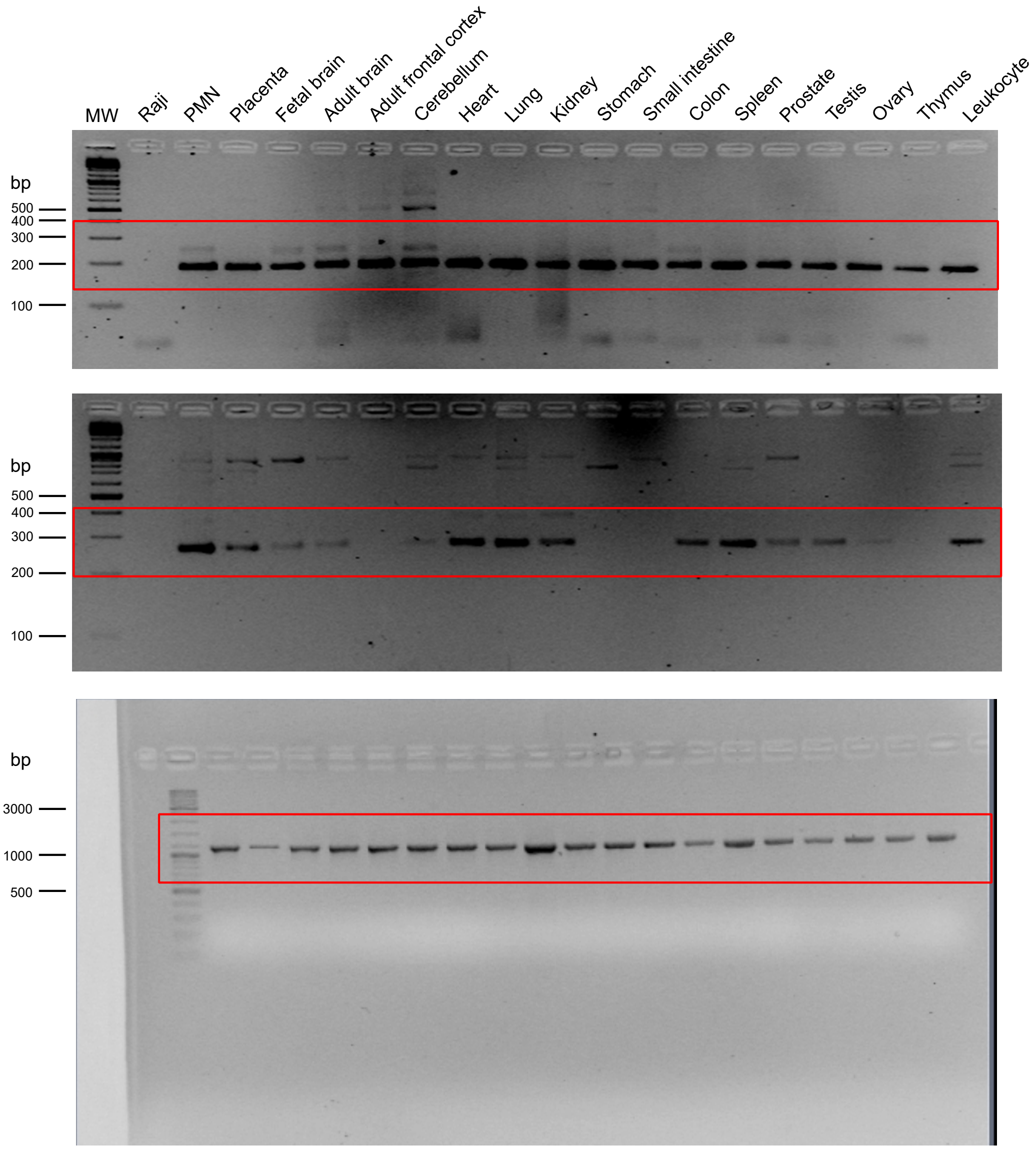
MW
Raji
PMN
Placenta
Fetal brain
Adult brain
Adult frontal cortex
Cerebellum
Heart
Lung
Kidney
Stomach
Small intestine
Colon
Spleen
Prostate
Testis
Ovary
Thymus
Leukocyte
bp
500
400
300
200
100
bp
500
400
300
200
100
bp
3000
1000
500

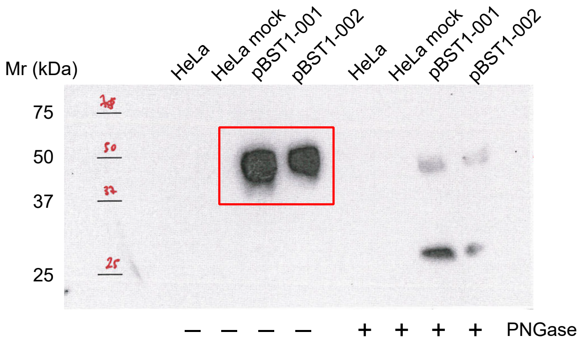
Mr (kDa)
HeLa
HeLa mock
pBST1-001
pBST1-002
HeLa
HeLa mock
pBST1-001
pBST1-002
75
50
37
25
PNGase

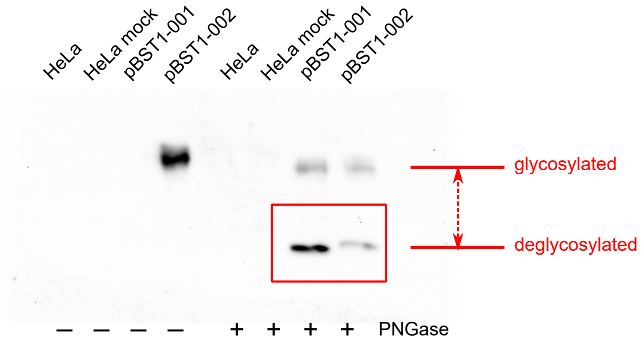
HeLa
HeLa mock
pBST1-001
pBST1-002
HeLa
HeLa mock
pBST1-001
pBST1-002
glycosylated
deglycosylated
PNGase

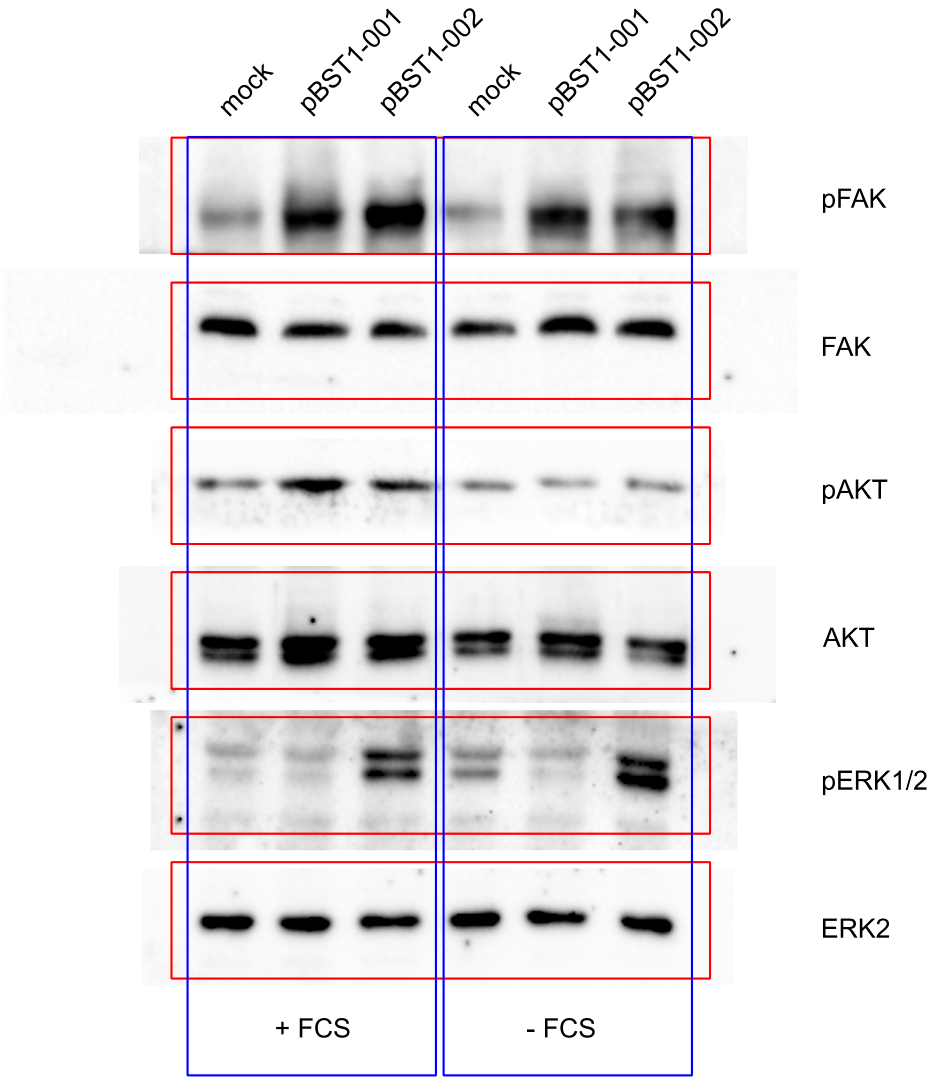
mock
pBST1-001
pBST1-002
mock
pBST1-001
pBST1-002
pFAK
FAK
pAKT
AKT
pERK1/2
ERK2
+ FCS
- FCS

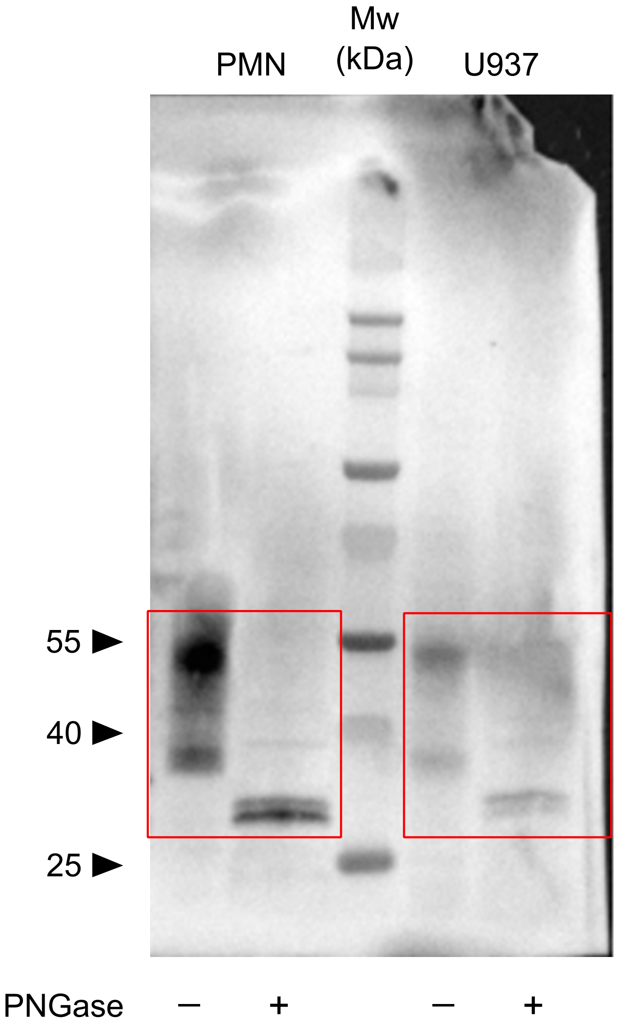
PMN
Mw
(kDa)
U937
55
40
25
PNGase
–
+
–
+
